# Utilizing 3D Models to Unravel the Dynamics of Myeloma Plasma Cells’ Escape from the Bone Marrow Microenvironment

**DOI:** 10.3390/cancers16050889

**Published:** 2024-02-22

**Authors:** Stefaan W. Verbruggen, Ciara L. Freeman, Fiona E. Freeman

**Affiliations:** 1 Digital Environment Research Institute, Queen Mary University of London, London E1 4NS, UK; 2 Center for Predictive In Vitro Models, School of Engineering and Materials Science, Queen Mary University of London, London E1 4NS, UK; 3 INSIGNEO Institute for In Silico Medicine, University of Sheffield, Sheffield S1 3JD, UK; 4 H. Lee Moffitt Cancer Center and Research Institute, Tampa, FL 33612, USA; ciara.freeman@moffitt.org; 5 School of Mechanical and Materials Engineering, Engineering and Materials Science Centre, University College Dublin, D04 V1W8 Dublin, Ireland; 6 Conway Institute of Biomolecular and Biomedical Research, University College Dublin, D04 V1W8 Dublin, Ireland; 7 Trinity Centre for Biomedical Engineering, Trinity Biomedical Sciences Institute, Trinity College Dublin, D02 PN40 Dublin, Ireland; 8 Department of Mechanical Manufacturing, and Biomedical Engineering, School of Engineering, Trinity College Dublin, D02 PN40 Dublin, Ireland; 9 Advanced Materials and Bioengineering Research Centre (AMBER), Royal College of Surgeons in Ireland and Trinity College Dublin, D02 YN77 Dublin, Ireland

**Keywords:** multiple myeloma, biological models, bone marrow, 3D printing, microfluidic devices, organ-on-a-chip

## Abstract

**Simple Summary:**

New treatments have made a big difference in helping people with multiple myeloma (MM) live longer. In fact, survival rates have almost doubled compared with treatments before 2000, thanks to these new and effective medications. This means more people with MM are surviving for more than 10 years after being diagnosed. However, because people are living longer, some unusual situations are arising, like cancer coming back in places outside the bone marrow, such as organs, lymph nodes, and even the central nervous system. To better understand and treat these new challenges, scientists are using advanced 3D models, especially looking at the bone marrow and other places where cancer may show up. This review identifies the best ways to use these 3D models, discusses the difficulties in recreating the complexity of the disease, and emphasizes how these advanced models can help fight MM.

**Abstract:**

Recent therapeutic advancements have markedly increased the survival rates of individuals with multiple myeloma (MM), doubling survival compared to pre-2000 estimates. This progress, driven by highly effective novel agents, suggests a growing population of MM survivors exceeding the 10-year mark post-diagnosis. However, contemporary clinical observations indicate potential trends toward more aggressive relapse phenotypes, characterized by extramedullary disease and dominant proliferative clones, despite these highly effective treatments. To build upon these advances, it is crucial to develop models of MM evolution, particularly focusing on understanding the biological mechanisms behind its development outside the bone marrow. This comprehensive understanding is essential to devising innovative treatment strategies. This review emphasizes the role of 3D models, specifically addressing the bone marrow microenvironment and development of extramedullary sites. It explores the current state-of-the-art in MM modelling, highlighting challenges in replicating the disease’s complexity. Recognizing the unique demand for accurate models, the discussion underscores the potential impact of these advanced 3D models on understanding and combating this heterogeneous and still incurable disease.

## 1. Introduction

Multiple myeloma (MM) is the second leading haematological malignancy in the United States [1]; a malignant neoplasm of plasma cells, which primarily affects the bone marrow. It remains incurable, with a high preponderance for clonal evolution and progressively shorter responses over time [2,3,4,5]. Significant improvements in disease management have increased survival for all patients with MM so that more patients are living longer and receiving anti-myeloma treatment for extended durations [6]. The progression of myeloma cells from bone marrow reliance to extramedullary proliferation is a significant phase in the disease’s development, presenting challenges in treatment due to the reduced responsiveness of these sites to the vast array of agents available for treatment [7]. Understanding the biological transitions of plasma cells from the bone marrow microenvironment to extramedullary sites is key to devising strategies for prevention and more effective treatment of extramedullary disease (EMD). Advancements in three-dimensional (3D) in vitro and in silico modelling hold great promise in assisting with unravelling key biology in myeloma research. These models can offer a more accurate representation of the myeloma microenvironment than traditional in vitro approaches, allowing for a deeper exploration of cellular dynamics and treatment responses. Therefore, delving into the current state of 3D models in myeloma is not only relevant but crucial for comprehending and addressing the complex biology hampering the complete eradication of the disease. This review provides an overview of the current 3D models of MM and establishes the crucial role played by 3D models, with a specific focus on the bone marrow microenvironment and extramedullary sites. We discuss the current state-of-the-art in MM modelling, highlighting challenges in replicating the disease’s complexity. Recognizing the unique demand for accurate models, the discussion underscores the potential impact of these advanced 3D models on understanding and combating this heterogeneous and still incurable disease.

## 2. Present Status of Therapeutic Advancements in Multiple Myeloma Treatment

The treatment landscape of MM has undergone significant transformations in recent years, marked by the introduction of novel therapeutic agents and strategies that have reshaped the standard of care (Table 1). The advent of proteasome inhibitors (PIs), immunomodulatory drugs (IMiDs), and monoclonal antibodies (mAbs) has expanded the therapeutic arsenal, offering improved outcomes and enhanced quality of life for patients [8,9]. Notably, the integration of these novel agents into frontline therapy, maintenance, and relapse settings has led to prolonged survival rates and deeper remissions [10]. Additionally, the emergence of chimeric antigen receptor T-cell (CAR-T) therapy represents a ground-breaking advancement, particularly for relapsed/refractory multiple myeloma (RRMM), showcasing remarkable efficacy in clinical trials and reproducible in real-world settings [11,12,13,14,15,16]. More recently, T-cell engaging (TCE) therapies have also been approved and are available in a similar population of patients with RRMM after exposure to greater than or equal to four prior lines of therapy [17,18,19]. However, despite these advances, challenges remain, including the management of multi-drug resistance and the treatment of high-risk subgroups. The development of extramedullary disease is increasingly common in those with progressively refractory disease, resulting in suboptimal depth and durability of responses from these novel therapies [7]. Furthermore, the heterogeneity in the disease continues to necessitate individualized treatment approaches, underscoring the importance of ongoing research and innovation. As such, the present status of MM treatment is one of dynamic evolution, with ongoing clinical trials and research efforts directed at further refining treatment protocols and discovering new treatment strategies capable of overcoming challenging biology.

### Extramedullary Disease in Multiple Myeloma: Unravelling the Intricacies and Clinical Implications

Extramedullary disease (EMD) has been heterogeneously defined in the literature, often encompassing both the growth of clonal plasma cells at anatomic sites distant from the bone marrow and also those that arise contiguous with osseous lesions that break through the cortical bone [20]. A recent consensus paper suggested that these should be split into two distinct entities based on their origin: (1) paraskeletal plasmacytomas (PSP), referring to those lesions that arise from focal bone lesions and disrupt the cortical bone, versus true (2) extramedullary plasmacytomas (EMPs), consisting of infiltrative collections of plasma cells in a variety of anatomical locations distant/separate from bone that have occurred as a result of hematogenous spread [21]. Studies on the incidence and the biology of EMD have generally been observational in nature, and the reported overall incidence across these newly defined subtypes is highly variable (Table 2).

**Figure 1:**
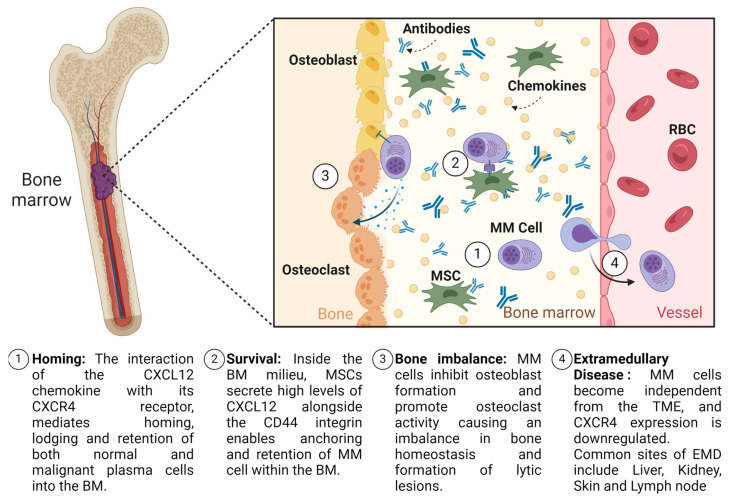
Schematic of the key events of MM disease progression. Abbreviations. MM: MM; RBC: red blood cell; BM: bone marrow; EMD: extramedullary disease; TME: tumour microenvironment. Created with Biorender.com (13th February 2024).

As plasma cells develop the capability to leave the supportive niche of the bone marrow microenvironment, key changes occur (Figure 1). These developments remain incompletely profiled due to the challenges in studying the trajectory of extramedullary disease from precursor to established metastatic spread. The current understanding supports the evolution of EMD as being facilitated by alterations in adhesion markers and signalling pathways. The detachment from the bone marrow microenvironment involves the downregulation of adhesion molecules like VLA-4 (Very Late Antigen-4) and integrins [23,24], which reduces their binding to stromal cells and the extracellular matrix, a process crucial for their entry into the bloodstream. Concurrently, the expression of chemokine receptors such as CXCR4 may be upregulated, facilitating migration towards new niches [25]. Finally, in circulation, plasma cells must survive without the support of the bone marrow stroma. This survival is often facilitated by the activation of signalling pathways like NF kappa B pathways, promoting cell survival and proliferation in the face of external stressors [26]. Upon colonizing extramedullary sites, myeloma cells might exploit the RAS/MAPK pathway for enhanced growth and survival capabilities, contributing to the aggressive nature of extramedullary disease [27,28]. The development of extramedullary disease may also be associated with the downregulation of key surface immunotherapy targets, which may also partly contribute to the suboptimal outcomes seen in patients with concomitant EMD treated with both CAR-T and TCE [29].

Plasmacytomas can potentially develop in any site of the body (Table 3) and can vary based on the disease stage. Available reports suggest that in newly diagnosed patients, where the reported incidence is generally considered to be much lower, EMPs most commonly affect the skin and soft tissues, whereas, at late relapse, sites of involvement more commonly include the liver, kidneys, lymph nodes, central nervous system (CNS), breast, pleura, and pericardium [7]. In terms of incidence in the relapsed setting, this appears to be increasing in the era of novel therapies. Prior reports suggested that EMD (the all-encompassing definition) affected up to ~30% of patients; however, this is possibly an underestimation. An autopsy series was able to find evidence of plasma cell infiltration at a variety of sites outside the marrow in approximately two-thirds of the patients studied [30]. Recent clinical trials of novel immunotherapies, including autologous T-cells transduced with a chimeric antigen receptor (CAR-T) and T-cell engaging therapies (TCE), in patients with triple-class exposed RRMM, suggest that the incidence is also higher. It ranges up to almost 50% in this growing and unique population [31]. These numbers may also underestimate the true incidence in these RRMM patients, as several clinical trials involving these novel therapies either limited or excluded patients with EMD. This decision was based on the observation that patients with EMD had inferior outcomes when treated with these novel therapies [32,33].

Investigating the evolution of EMD presents unique challenges. One of the critical tools moving forward is the development and utilization of appropriate 3D models. These models are indispensable for dissecting the complex interactions between myeloma cells and their ability to leave the bone marrow microenvironment, which is crucial in the pathogenesis of EMD. The rarity and heterogeneous presentation of EMD pose a substantial challenge in acquiring representative patient samples, and it is difficult to rely on these samples alone to study the development of the disease. Secondly, while mouse models are often a cornerstone of cancer research, they fall short in faithfully replicating the human occurrence of EMD, particularly due to differences in immune system functioning and tumour microenvironment interactions. This inadequacy limits our ability to generalize findings from these models to human patients. Lastly, and perhaps most critically, the biology of extramedullary myeloma may differ depending on the site of involvement. EMD can occur in a variety of tissues, each with its unique microenvironment, and these differences may profoundly impact the behaviour of myeloma cells. A 3D model that is capable of adequately recapitulating these varied environments is essential for a comprehensive understanding of EMD biology.

## 3. Three-Dimensional Models of Multiple Myeloma

### 3.1. In Silico MM Bone Marrow Models

The advent of large-scale computing power in recent decades has generated a revolution in computational, or in silico, modelling power. This has brought singular advantages to the study of a range of cancers. To date, the focus of MM research has been bioinformatics modelling of large genomics and transcriptomics datasets, as well as pharmacokinetic and pharmacodynamic (PKPD) simulations of drug interactions. These have led to significant findings, including identifying novel signalling pathways [38,39], genetic risk factors [40,41], biomarkers [42,43], RNA interactions [44,45,46,47], and oncogenic inflammation and microenvironments [48,49]. Similarly, these large datasets can be used to develop models of the effects and side effects of drug interventions [50] by modelling the signalling interactions of different cell types [48]. While these studies all applied mathematical modelling to large experimental datasets, perhaps the greatest strength of computational modelling is the ability to generate a 3D virtual tumour microenvironment and demonstrate changes in cell behaviours and interactions in response to changing biochemical conditions. This was recently achieved using finite element modelling, enabling the study of cell motility, proliferation, differentiation, and apoptosis, along with interactions with native bone cells and cancer-associated cells [51]. Computational models of all types can be limited by the quality of input data and idealized representations of the in vitro environment [52]. Nonetheless, further developments using these methods could facilitate linkages with macroscale models of bone lesions, such as in vertebrae, resulting in patient-specific predictions of disease progression in 3D.

### 3.2. Three-Dimensional Tissue-Engineered MM Models

The use of tissue engineering techniques to create 3D models of MM offers valuable platforms for in-depth research and innovative therapeutic investigations. These models utilize patients’ own bone marrow (BM) cells and aim to better replicate the complexity and heterogeneity in the BM microenvironment. By incorporating the unique cellular and spatial interactions within the MM microenvironment, these 3D tissue-engineered models have the potential to provide a more accurate representation of the disease and serve as valuable platforms for drug discovery and testing. 

**Figure 2:**
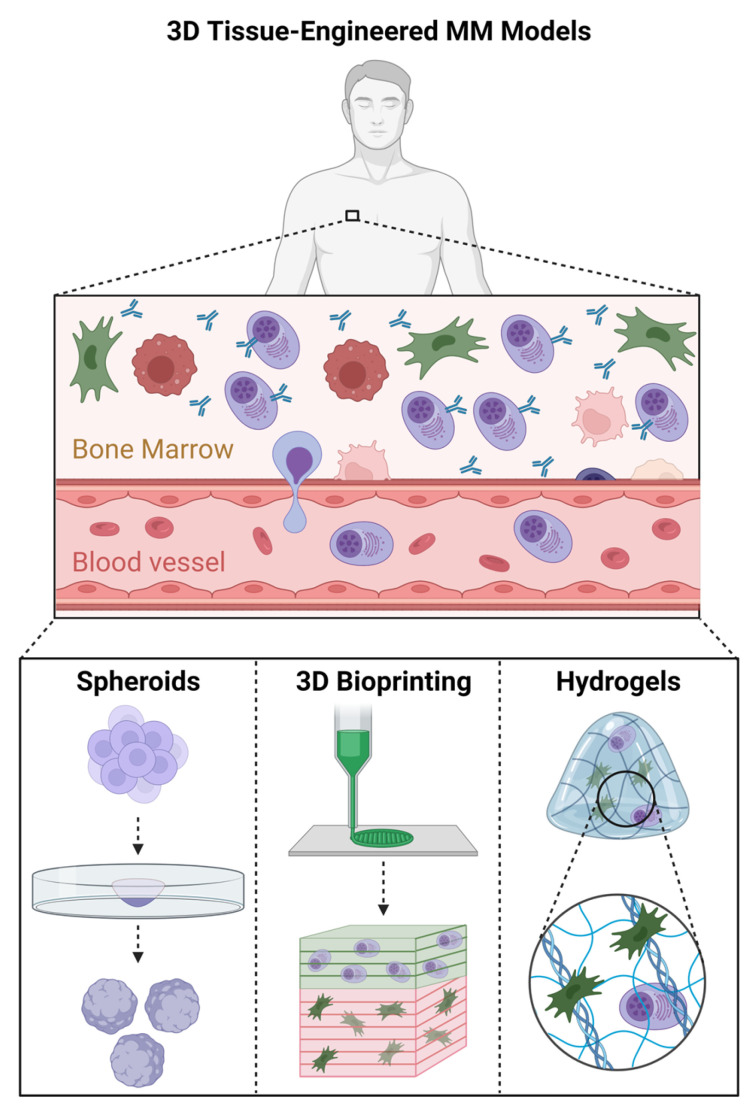
Three-dimensional tissue-engineered MM models: 3D cellular spheroid models, 3D bioprinting models, and 3D hydrogel models. Created with Biorender.com (15th February 2024).

The models can be categorized into three distinct groups: 3D cellular spheroid models, 3D bioprinting models, and 3D hydrogel models (Figure 2). Here, we review the advantages and limitations of the presently available tissue-engineered three-dimensional models for MM (Table 4). Additionally, we explore the ongoing challenges and provide insights into strategies for achieving a more biomimetic and precise MM BM model.

#### 3.2.1. Three-Dimensional Spheroid Models of MM

Three-dimensional cellular spheroid models have gained significant attention in the field of cancer research, including the study of MM and its extramedullary manifestations. Scaffold-free 3D cellular spheroid models provide a unique advantage by allowing cells to self-aggregate and form spheroids that mimic the architecture and behaviour of tumours more closely compared with traditional 2D culture systems [53]. These spheroids can be generated by aggregating cancer cells in suspension, promoting cell–cell interactions, and enabling the secretion of their own extracellular matrix (ECM). This self-secreted ECM provides structural support and mimics the microenvironment in which the cells reside, thereby enhancing the physiological relevance of the model.

Previous studies have demonstrated the utility of bone marrow organoids in supporting the engraftment, survival, and proliferation of myeloma cells in vitro for up to 12 days [54,55]. However, there are certain limitations to using 3D cellular spheroid models for studying myeloma. One major drawback is the lack of a vascular network or the integration of the perivascular niche within the model. Vascularization and the perivascular niche are crucial when modelling myeloma in vitro. They play important roles in disease progression, angiogenesis, and the interaction between myeloma cells and the microenvironment. This also means that these models do not necessarily replicate the nutrient concentration equilibrium in the medium that is maintained in vivo by various physiological processes. Incorporating these elements into models allows researchers to study their impact on disease behaviour and therapeutic interventions. This interaction is not fully recapitulated in the simplified 3D cellular spheroid models, limiting their ability to truly model the complexity of the tumour microenvironment.

Furthermore, the interaction between myeloma cells and the endosteal bone lining, which is a critical aspect of myeloma biology, has not been extensively investigated in previous studies utilizing 3D cellular spheroid models. The endosteal niche provides a specialized microenvironment that influences myeloma cell behaviour, including adhesion, migration, and drug resistance. Therefore, incorporating the endosteal component into the model would enhance its physiological relevance and allow for a more comprehensive understanding of myeloma pathogenesis.

#### 3.2.2. Bioprinted MM Bone Marrow Models

Bioprinting, a form of 3D printing, offers numerous benefits over traditional tissue engineering methods, facilitating the creation of highly sophisticated and realistic 3D models that closely mimic the complex nature of the disease. One of the key advantages of bioprinting when developing MM models is the ability to generate structures with distinct zones and multiple cell types within the same model. Bioprinters can precisely deposit different types of cells and biomaterials in specific locations, allowing for the recreation of the intricate cellular and spatial interactions present in the MM microenvironment. This capability enables researchers to study the dynamic interplay between various cell populations and investigate their contributions to disease progression.

Previous studies utilising 3D bioprinting have successfully modelled critical aspects of the MM microenvironment, including the perivascular niche [56], endosteum/bone lining-MM interaction [56,57], and the human bone marrow microenvironment [57]. These models have shed light on the essential role that the perivascular niche plays in supporting myeloma cells and influencing disease progression [56]. By incorporating relevant cell types, such as endothelial cells and mesenchymal stromal cells, along with myeloma cells, bioprinting allows researchers to examine the spatial distribution and functional interactions within these niches.

However, despite these advancements, it is important to note that existing bioprinted MM models often lack naturally occurring haematopoiesis and immune system interactions. The bone marrow microenvironment is a complex ecosystem that involves various components, including hematopoietic stem cells, immune cells, and stromal cells. These interactions are crucial in shaping the tumour microenvironment and influencing disease behaviour. While bioprinting has enabled the creation of sophisticated models, incorporating these additional components poses a significant challenge.

Future research endeavours should aim to integrate haematopoiesis and immune system interactions into bioprinted MM models. This would involve the incorporation of a more diverse microenvironment, in particular, tumour infiltrating macrophages, stromal cells, T cells and natural killer cells, to better mimic the natural complexity and intercellular crosstalk of this complex microenvironment. This would allow for a greater understanding of the potential interactions that could be targeted, by profiling these interactions and determining the key roles in disease progression.

#### 3.2.3. Hydrogel-Based MM Bone Marrow Models

Three-dimensional hydrogel models are the most common approach for studying MM. These models provide a more physiologically relevant environment for studying MM compared with traditional 2D cultures. Furthermore, the inclusion of perivascular components such as endothelial cells renders them a more advanced alternative to the 3D spheroid model. Various naturally derived hydrogels, such as Matrigel [55,58,59,60,61], collagen [55,58,60,61,62,63], fibronectin [60,61], hyaluronic acid [64], fibrin [65,66], and gelatin [67], have been used to generate 3D hydrogel models for MM. The majority of these studies utilised a co-culture system incorporating MM cells together with mesenchymal stromal cells (MSCs). MSCs play a crucial role in the bone marrow microenvironment and contribute to MM cell growth and survival in vivo [68]. By including MSCs in the hydrogel model, researchers aim to recreate the supportive niche provided by these cells. These studies demonstrated, similar to in vivo observations, that the addition of MSCs within the 3D hydrogel system enhanced MM cell viability [58,62,63] and increased CXCR4 expression on MM cells [62].

The perivascular niche plays a critical role in the myeloma tumour microenvironment, influencing tumour cell behaviour, angiogenesis, and therapeutic responses. In recent studies, the successful modelling of the perivascular niche has been achieved within 3D hydrogel models [58,59,66]. Perivascular components, such as endothelial cells and pericytes (MSCs), are integrated into the hydrogel, modelling the specialized microenvironment surrounding blood vessels in the bone marrow [58,59,66]. These models demonstrated the essential role the perivascular niche plays in MM cell survival and proliferation as MM cells continuously proliferated and remained viable in long-term in vitro cell culture (up to 28 days) [59]. These long-term cultures allow for the evaluation of drug responses and the investigation of disease progression mechanisms.

Additionally, the integration of endosteal components [59,60,61,66,69,70], including osteoblasts, osteoclasts, and the bone mineral matrix, has been accomplished to examine the interaction between MM cells and the bone lining within these models. It is well documented that the osteoblastic niche within the bone marrow plays a crucial role in maintaining the dormancy of MM cells, whereas osteoclasts contribute to the reactivation of MM cells [68]. Hence, it is crucial to model this interaction in order to accurately replicate the tumour microenvironment. These studies demonstrated that MM cells cultured in 3D are dependent on their STAT3 activity [60] for superior cell viability compared with conventional culture. The prolonged longevity of MM cells in 3D allows for long-term gene manipulations and/or drug treatment, enabling the study of MM in a more realistic manner.

The interaction between the immune system and the myeloma tumour microenvironment is a complex process involving immune cell infiltration, immune cell-mediated cytotoxicity, and the modulation of immune responses, which can significantly impact disease progression and treatment outcomes. As a result, one study expanded the complexity of their 3D hydrogel models by including immune cells [59]. In this study, T cells were introduced into the 3D hydrogel model, enabling their infiltration and cytotoxic attack on MM cells within the developed model. This model has the potential to examine immune cell-mediated cytotoxicity and more novel immunotherapeutic approaches within a more relevant 3D microenvironment.

Yet, it is important to note that myeloma is a liquid cancer that primarily resides within the bone marrow, which necessitates the inclusion of a fluidic component within these models to mimic the disease more accurately. While 3D hydrogel models provide an improved representation of the myeloma microenvironment, none of the proposed hydrogel systems truly mimic the mechanical environment of the bone marrow cavity, and none to date can effectively capture the events that predispose to the development of EMD. The fluid flow and mechanical forces present in the marrow microenvironment can influence myeloma cell behaviour and drug response, highlighting the need for further advancements in modelling these aspects.

**Table 4:** Available tissue-engineered three-dimensional models for MM.

3D Model	Cells	Biomaterials	In Vitro Viability	Advantages	Limitations	Refs
Spheroids	CD14+ Monocytes and MM cells	Matrigel	3 days	Scaffold-free allows for cells to self-aggregate and secrete own ECM. Modelled the protective effect of the microenvironment on MM cells. Addition of monocytes to the co-culture promoted MM cell viability, proliferation, and drug resistance.	Lacking natural occurring haematopoiesis and immune system interactions. Does not model the endosteum-MM interaction. Does not model the perivascular niche.	[54]
Spheroids + hydrogel	iPSC, MSCs, MM cells	Matrigel and Collagen Type I	12 days	Bone marrow organoids support the engraftment, survival, and proliferation of MM cells.	Lacking natural occurring haematopoiesis. Does not model the endosteum-MM interaction. Does not model the perivascular niche.	[55]
Bioprinting	O-MSCs, MSCs and EPCs, and CD138+ MM cells	Matrigel and Calcium Phosphate Cement	28 days	Modelled the perivascular niche. Modelled the endosteum–MM interaction. Increased the proliferation of MM cells.	Lacking natural occurring haematopoiesis and immune system interactions.	[56]
Bioprinting	MM cells and fibroblasts	Gelman, nHA, Alginate, and PEGDA	9 days	Three-dimensional-bioprinted MM model that emulates the human bone marrow. MM cells can be maintained within 3D-bioprinted construct with good viability for up to 7 days.	Lacking natural occurring haematopoiesis and immune system interactions. Does not model the perivascular niche.	[57]
Hydrogel	MSCs and EPCs, and CD138+ MM cells	Matrigel	14 days	Modelled the perivascular niche. Interactions between the MM cells and MSCs improved the survival of the MM cells. The porous hydrogel system supported the passive diffusion of therapeutic nanoparticles.	Does not model the endosteum-MM interaction. Lacking natural occurring haematopoiesis and immune system interactions.	[58]
Hydrogel	MSCs, CD138+-selected MM patients’ cells, and patient-derived plasma	Collagen-I	5 days	Interactions between the MM cells and MSCs improved the survival of the MM cells and increased CXCR4 expression on MM cells.	Lacking natural occurring haematopoiesis and immune system interactions. Does not model the endosteum-MM interaction. Does not model the perivascular niche.	[58,62,63]
Hydrogel	MM cells	Matrigel, Fibronectin, and Collagen Type IV	6 days	Modelled the endosteum–MM interaction. Activation of STAT3 was observed in 3D cells but not in 2D cells.	Utilised MM cell lines. Lacking natural occurring haematopoiesis and immune system interactions.	[60]
Hydrogel	MSCs and MM cells	Parametric	7 days	Co-culture between the MM cells and MSCs improved the survival of the MM cells. Co-culture in increased CXCR4 expression on MM cells. Modelled drug resistance.	Lacking natural occurring haematopoiesis and immune system interactions.	[62]
Hydrogel	MM cells	Matrigel, Fibronectin, and Collagen Type I	25 days	Modelled the endosteum–MM interaction. Long-term culture of MM cells.	Lacking natural occurring haematopoiesis.	[61]
Hydrogel	MM cells	Hyaluronic Acid	21 days	HA hydrogels with medium mechanical stiffness (~500 kDA) could support MM cell survival. Long-term culture of MM cells.	Lacking natural occurring haematopoiesis and immune system interactions. Does not model the endosteum–MM interaction. Does not model the perivascular niche.	[64]
Hydrogel	MM cells, MSCs, and EPCs	Fibrinogen	7 days	The 3DTEBM cultures allowed for the proliferation of MM cells. Recreated 3D aspects observed in the bone marrow niche (such as oxygen and drug gradients). Modelled drug resistance.	Lacking natural occurring haematopoiesis and immune system interactions. Does not model the endosteum–MM interaction.	[65]
Hydrogel	O-MSCs, HUVECs, and MM cells	Silk Fibroin	1 month	Modelled the bone–MM interaction. Long-term culture of MM cells. Modelled the perivascular niche. Modelled drug resistance.	Lacking natural occurring haematopoiesis and immune system interactions.	[66]
Hydrogel	MM and MSCs	PGMA_52_–PHPMA_122_ diblock copolymer	7 days	Modelled the paracrine interaction between MSCs and MM cells. Identified that IL-6 and IL-10 play a critical role in sustaining MM cell proliferation.	Utilised MM cell lines. Lacking natural occurring haematopoiesis and immune system interactions. Does not model the endosteum–MM interaction. Does not model the perivascular niche.	[71]
Hydrogel	MSCs and MM cells	Gelatine Sponge	3 days	MM cells after contact with BMSCs in 3D cultures produced more sIL-6R than in the classic 2D cultures.	Lacking natural occurring haematopoiesis and immune system interactions. Does not model the endosteum–MM interaction. Does not model the perivascular niche.	[67]

Abbreviations. MM: multiple myeloma; iPSCs: induced pluripotent stem cells; MSCs: mesenchymal stromal cells; MSC: O-MSCs: osteogenically differentiated mesenchymal stromal cells; EPCs: endothelial progenitor cells; nHA: nanohydroxyapatite; PEGDA: polyethylene glycol diacrylate; HUVECs: human umbilical vein endothelial cells.

### 3.3. Microfluidic and Organ-on-a-Chip Models of MM

Microfluidic devices, small engineered plastic systems in which fluid flow and cells can be precisely controlled, have emerged as valuable tools in many fields of cancer research, offering unprecedented opportunities to investigate various aspects of the disease in different organs [72]. Given the nature of MM as a liquid cancer, these transparent microfluidic chambers are ideally suited to observe the effects of biological, mechanical, and chemical interventions. Furthermore, as the founding technology of organ-on-a-chip research, microfluidic devices allow for the co-culture of various cell types and offer precise control over key environmental factors such as mechanical forces, chemical gradients, and cell–cell interactions. This enables the faithful recapitulation of tissue-level organization, cell fate determination, and even the formation of functional organs within a chip. In MM and EMD research, this has allowed for a range of investigations to be performed.

#### 3.3.1. Circulating Tumour Cell Analysis

Microfluidic devices have been used for decades to capture and analyse circulating tumour cells (CTCs) in MM patients’ blood samples. Initially applied to investigate CTCs under flow, these devices can be adapted to include specific surface markers or physical properties of CTCs to isolate and characterise them. By studying CTCs, researchers can gain insights into disease progression, monitor treatment response, and investigate the mechanisms underlying metastasis and drug resistance.

The first application of this technology to MM was carried out by Pilarski and colleagues in the 2000s, in a study that applied microfluidic devices to sort cells and selectively apply electrophoresis, detecting PCR product amplified transcripts or DNA from individual cells within patient samples [73,74]. In doing so, this work provided the first demonstration of the accurate and versatile detection of molecular signatures in individual myeloma cells, showing the value of microfluidic devices for monitoring response to therapy, detecting residual cancer cells that mediate relapse, and evaluating prognosis.

**Figure 3:**
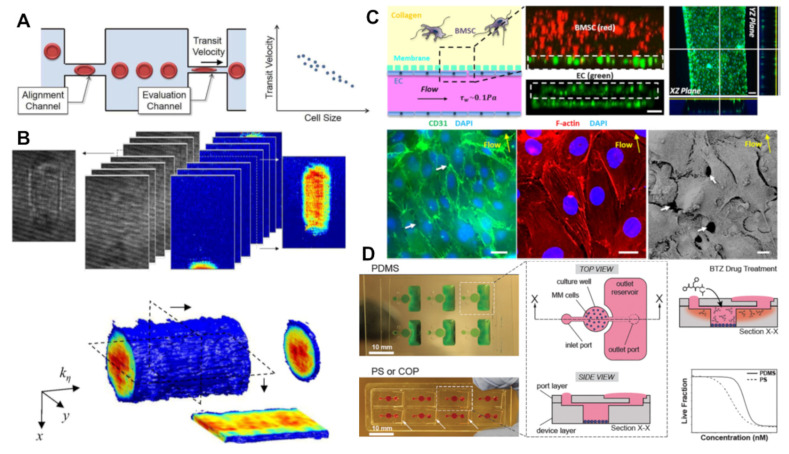
Microfluidic-based systems for MM analysis and modelling: (**A**) Measurement of red blood cell shape when flowing through microfluidic channels as a diagnostic biomarker [75]. (**B**) Three-dimensional holographic measurements of continuously flowing circulating tumour cells [76]. (**C**) An organ-on-a-chip model of the tumour microenvironment within the bone marrow niche, including bone marrow stem cells (BMSCs) and endothelial cells (ECs) [79]. The white arrows point to open gaps between ECs. (**D**) Testing of the efficacy of bortezomib (BTZ) drug treatments in microfluidic chips composed of multiple different chip materials, polydimethylsiloxane (PDMS), polystyrene (PS), and cyclo-olefin polymer (COP) [80].

A number of interesting developments occurred in the following decade, which used the transparent nature of the chips with the precise control of mechanical forces to analyse cells from MM patients in novel ways. Given the scarcity of cancer cells within blood samples, Tsai et al. instead focused on the most abundant blood cell type, investigating whether the mechanical properties of red blood cells in flow were significantly different in MM patients (Figure 3A). They found that, compared with healthy patients, MM patients may present significantly stiffer red blood cells and that this could indeed be used as a diagnostic biomarker [75]. At the same time, Sung and colleagues developed a method that took advantage of the refractive index of cells to generate 3D holographic measurements of continuously flowing cells [76]. They found that this represented a useful label-free tool for quantifying CTC mechanics and for the rapid identification of CTCs within samples (Figure 3B). Another approach, which applied mechanical forces to directly trap cells, was developed by Ouyang et al., who built micropillars into their microfluidic devices to allow for the trapping of individual CTCs [77]. By correlating the trapping of these cells to serum protein levels, the researchers posited a diagnostic potential. They later demonstrated that the device is as effective as serum biomarkers in determining the effects of treatments on patients [78].

While the above methods largely rely on the inherent mechanical control and imaging advantages provided by microfluidic chips, additional advances can be made by combining them with biochemical assays. Quassimeh et al. [81] coated the surfaces of microfluidic channels with CD138 antibodies to selectively capture CD138+ MM cells. Combined with herring-bone geometries to increase the surface contact with cells, this device demonstrated capture efficiencies of up to 70% in patient samples, identifying MM patients with similar success to serum assays and indicating its potential as a liquid biopsy. Conversely, another group later used microfluidic channels to separate out cells, depleting the sample of CD45+ cells that usually predominate and dilute the CD138+ cells [82]. This technique allowed them to increase the concentration of CD138+ cells greater than 3-fold, and, in doing so, they improved the detection of key biomarkers. Finally, in a further development of the coated herringbone concept, Liu et al. coated a similar device in which the ligand for CD138+ was tuneable [83]. This allowed MM cells to be trapped and also selectively released back into the sample in response to a given stimulus, thus allowing for cells expressing different phenotypes to be sorted out and analysed separately. Most recently, these methods have been allied with other techniques to develop an FDA-approved method for CTC detection and genomic profiling in the clinic, for both diagnostic and prognostic purposes [84,85].

These studies demonstrate the ability of microfluidic chips to precisely control individual cells in flow, which, coupled with recent developments in next-generation sequencing, provides novel opportunities to identify new pathways and markers governing cell fate. Performing simultaneous genome and transcriptome profiling on these circulating tumour cells has been shown to be feasible and highly prognostic, confirming the validity of this approach [84,86].

#### 3.3.2. Tumour Microenvironment Modelling

Microfluidic devices enable the creation of 3D organ-on-a-chip models that mimic the complex tumour microenvironment of MM. These devices can incorporate cancer cells, stromal cells, and extracellular matrix components to replicate the cellular interactions and physical cues present in the bone marrow niche. By recreating the tumour microenvironment, researchers can study the effects of different factors on MM development, progression, and drug resistance. In mimicking the complex architecture and functionality of human organs at a microscale, organ-on-a-chip models offer an innovative approach to simulate the transition from the bone marrow microenvironment to the development and seeding of extramedullary disease. These devices can accurately replicate the intricate cellular interactions and microenvironments of both the bone marrow and extramedullary sites, providing a dynamic platform to study the migration of myeloma cells and their interaction with different tissue environments. This approach presents an unprecedented opportunity to closely observe and analyse the mechanisms that drive myeloma cells to leave their primary niche and establish secondary disease sites. Consequently, microfluidic 3D organ-on-a-chip models hold significant potential for advancing our understanding of MM progression and for testing new therapeutic strategies targeting both the bone marrow microenvironment and extramedullary disease sites.

Zhang et al. generated the first 3D model of the tumour microenvironment in the bone marrow niche (see Figure 3C): a device incorporating human MSC and osteoblast cell lines co-cultured with primary human MM cells [87]. They used this model to measure the degree of expansion of tumour cells from each patient and then further developed it to provide the first organ-on-a-chip model that could open the way to (1) testing personalized therapeutics for MM patients; (2) evaluating new drugs without the need for costly animal models; and (3) studying the biology of MM, and in particular, the mechanisms responsible for drug resistance and relapse [88]. This model was later adapted further to mimic additional aspects of the bone marrow environment, including sinusoidal circulation, sinusoidal endothelium, and stroma [79]. They found that MM cells induced a less organized and loosely connected endothelium, the widening of endothelial cell junction pores, and increased permeability through endothelial cells.

A similar model was built by Moore et al., who used a static array to co-culture bone marrow MSCs with MM cells to investigate cell–cell interactions via three key cytokines (IL-6, VEGF, and TNFa) in conditioned media [80]. Similarly, Sarkar et al. co-cultured MM cells with immune cells (dendritic cells and T cells) and found that T cell activity against MM cells was partially mediated by interferon-gamma [89]. Separately, Pak et al. co-cultured patient-derived CD138+ MM cells with companion CD138− stained cells from the same patient samples and found that this improved the prognostic effect of the microfluid device [90].

While most organ–chip models generate tumour microenvironments inside the chip channels, a couple of innovative approaches have applied the inherent fluid dynamics and control of microfluids at this scale to generate unique microenvironments. A particularly innovative design developed by de Groot et al. used the inherent surface tension in culture medium to generate an open hanging droplet model in which two wells were connected via a microfluidic channel [91]. In this way, they were able to add substrates and reagents at will, without inducing the high shear forces that can sometimes be problematic in microfluidic channels. Another group enlisted micro-manipulation capabilities to construct microenvironments within individual droplets [91]. In doing so, they created suspended droplets containing micro-niches of MM cells and MSCs.

#### 3.3.3. Drug Screening and Personalized Medicine

A further advantage of microfluidic devices is the capability to facilitate high-throughput drug screening for MM. By miniaturizing experimental assays and incorporating patient-derived cells, researchers can evaluate drug efficacy and toxicity with greater precision. Microfluidic platforms also allow for the testing of combination therapies and personalized medicine approaches, where patient-specific characteristics can be incorporated to identify the most effective treatment strategies for individual patients.

An early study by Khin et al. built a preclinical assay by combining microfluidic devices with a computational model to successfully predict responses of both cell lines and cancer patient cells to bortezomib and melphalan treatments [92]. Similarly, a number of the tumour microenvironments mentioned in the preceding section were later applied to test and predict drug resistance. Silva et al. developed an innovative ex vivo model for predicting clinical responses in MM, combining chemosensitivity assays in a hydrogel matrix with computational modelling [63,93]. This approach is significant as it allows for the analysis of drug efficacy in primary MM cells, including patient cells, patient serum, and some elements of the tumour microenvironment. One of the major advantages of this method is its detailed characterization of tumour chemosensitivity and integration with mathematical models, enabling the accurate prediction of clinical responses. This approach is limited in that it attempts to translate short-term assay results into long-term clinical outcomes, and the stroma utilized is not patient-specific. However, these are limitations that this methodology begins to bridge through mathematical modelling. Pak et al.’s 2015 model was tested by co-culturing it with companion cells to assess drug resistance, which correctly predicted increased efficacy in the presence of co-culture [90]. The model developed by Moore et al., as mentioned above [50,80], was later applied to develop a key study that compared PDMS as a chip material with polystyrene (PS) and cyclo-olefin polymer (COP) (Figure 3D), and a significant decrease in relative drug activity was found in PDMS chips [80]. This study demonstrated that the adsorption properties of PDMS are a significant limitation in using chips to test for certain drugs and suggested caution as the field progresses. Finally, in follow-up work by Sarkar et al. using the tumour microenvironment model described above [89], the team developed a droplet system showing enhanced activity by natural killer cells [94].

Overall, microfluidic devices have revolutionized MM research by offering precise control over experimental conditions, enabling the modelling of the tumour microenvironment and facilitating high-throughput analysis. By leveraging these devices, researchers can gain valuable insights into disease mechanisms, drug response, and personalized treatment strategies, ultimately driving advancements in the field and improving patient outcomes.

## 4. Concluding Remarks

The remarkable progress in treating myeloma over the past two decades has significantly extended survival rates and created a growing population of long-term survivors. However, the contemporary clinical landscape presents new challenges, notably the trend towards more aggressive relapse phenotypes, including the emergence of EMD and highly refractory aggressive clones. This review emphasizes the urgent need for advanced models to profile the evolution of MM, particularly its development to independence outside the bone marrow. Additionally, standardized culture methods and refined readout techniques will be key to the development of the field of research [95]. The exploration of state-of-the-art 3D models, especially in the context of the bone marrow microenvironment and the later development of extramedullary sites, underscores the complexity of replicating MM’s heterogeneous nature. The potential of these 3D models to accurately mimic the disease is invaluable, offering profound insights into MM’s progression and aiding in the development of innovative treatment strategies. Historically, drug testing has predominantly focused on small molecules, utilizing simpler in vitro models that fail to capture the complex cellular interactions occurring in vivo. However, the multi-cellular complexity of 3D models more closely mimics the intricate biological environment where myeloma evolves, offering a more physiologically relevant setting for evaluating and predicting responses to more novel and innovative immunotherapies, including CAR-T cell therapy and T cell redirecting bispecific antibodies [96]. This fidelity is crucial for assessing the nuanced interactions between therapeutic agents and the tumour microenvironment, enabling a more precise evaluation of their efficacy and potential side effects. In addition, given the multitude of immunotherapy targets and the potential combination therapies that are approved for MM, the traditional approach of conducting extensive clinical trials for each possibility becomes untenable [97]. A well-developed in silico model, particularly one based on advanced 3D constructs, could dramatically accelerate research by simulating a vast array of treatment combinations [98], thereby streamlining the identification of the most promising therapeutic strategies without the need for countless human trials. As we strive to conquer diseases that have eluded cure, the continuous evolution and refinement of 3D models are essential. They hold the promise of ushering in a new era of personalized medicine, where treatments are not only more effective but also tailored to the individual’s unique biological landscape, thereby enhancing the likelihood of successful outcomes.

## Figures and Tables

**Table 1:** Overview of the current therapeutic agents available for patients with MM.

IMiDs	Proteasome Inhibitors	Chemotherapy Anthracyclines	Chemotherapy Alkylators	Steroids	mABs	Other Small Molecules	Complex Immunotherapies
Thalidomide (Thalomid)	Bortezomib (Velcade)	Adriamycin	Cyclophosphamide (Cytoxan)	Dexamethasone	Daratumumab/ anti-CD38 (Darzalex)	XPO1 inhibitor (Selinexor)	BCMA targeting ADCs (belantamab); CAR-T (ide-cel/cilta-cel); TCE (teclistamab, elrantamab).
Lenalidomide (Revlimid)	Carfilzomib (Kyprolis)	Doxil (liposomal doxorubicin)	Bendamustine	Prednisone	Isatuximab /anti-CD38 (Sarclisa)		GPRC5D targeting TCE (talquetamab).
Pomalidomide (Pomalyst)	Ixazomib (Ninlaro)		Mephalan		Elotuzumab/ anti-SLAMF7 (Empliciti)		

IMiDs: immunomodulatory drugs, mAbs: monoclonal antibodies, BCMA: B-cell maturation antigen, ADC: antibody–drug conjugate, CAR-T: chimeric antigen receptor T-cell therapy, TCE: T-cell engaging therapy, GPRC5D: G protein-coupled receptor, class C, group 5, member D, SLAMF7: signalling lymphocyte activation molecular family 7.

**Table 2:** Plasmacytomas in MM: incidence at diagnosis and at relapse [11,21,22].

	Paraskeletal Plasmacytomas (PSP), %	Extramedullary Plasmacytomas (EMP), %
At diagnosis	7–34.4	1.75–4.5
At relapse	6–34.2	3.4–48

**Table 3:** Most common sites of extramedullary involvement.

Site of Extramedullary Disease	Reported Incidence (%)	References
Skin/muscle	24	[34,35]
Pleura/lungs	12	[35,36]
Lymph nodes	10–23	[30,35]
Liver	9–28.8	[30,35]
Central nervous system (CNS)	3–6	[35,37]
